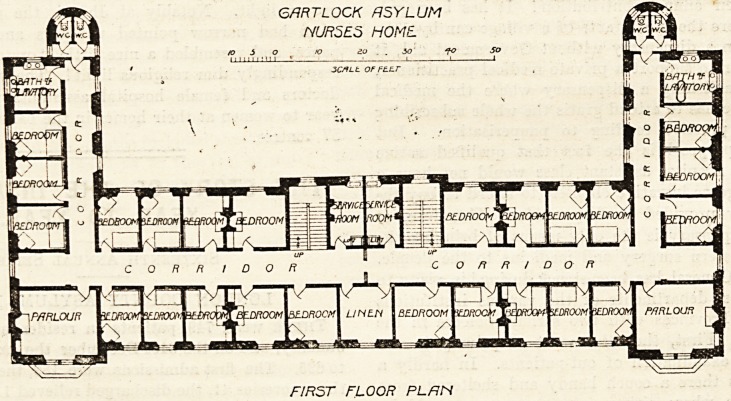# Nurses' Home: Glasgow District Asylum at Gartloch

**Published:** 1904-09-17

**Authors:** 


### HOSPITAL ADMINISTRATION.

### CONSTRUCTION AND ECONOMICS.

### NURSES' HOME: GLASGOW DISTRICT ASYLUM AT GARTLOCH.

DURING the last twenty years there has been a very considerable increase in the number of night nurses in our county and district asylums, and hence there is not the same necessity as there was at one time that all the day nurses should sleep close to their respective wards in case of being called up during the night. Nurses' homes are therefore springing up as adjuncts to our public asylums, and to obtain perfect quietness for ten hours out of the twenty-four must be one of the greatest boons which has lately fallen in way of our asylum nurses.

The home at the Gartloch Asylum is one of the most recently erected, and therefore we reproduce the ground and the first-floor plans. It is, however, a three-story building, but the second floor resembles the first floor in all particulars except that it is so arranged that one of its sections is set apart for the domestics and is reached by a separate staircase, while the other section is arranged so as to ensure quietness during the day, and is given up to the night nurses.

The plan is extremely simple. It consists of a centre and two wings, the latter being put on at right angles to the former. In the centre of the block is the entrance hall, and from this a wide corridor runs along the whole length of the central portion of the building and divides it longitudinally. The front part contains the matron's rooms, parcel room, stranger's room, two parlours, and four bed-sitting-rooms. The parlours occupy the extreme ends of the central part to the front. At the back of the corridor are the kitchen with its lifts, the staircases, cloak-rooms, and four bed-sitting-rooms. The doors of the parlours are exactly opposite the corridors giving access to the rooms of the wings. In each of these wings are three bedrooms, bathroom, and two closets, the latter being properly cut off from the corridor by a cross-ventilated passage. Practically the first floor differs from the ground floor only in certain minor points. For instance, the space over the entrance hall is here a linen-room; that over the kitchen is divided into two service-rooms; and bedrooms are placed over the cloak-rooms.

GROUND FLOOR PLAN.

J. THOMSON AND SANDILANDS

ARCHITECTS

241 WEST GEORGE ST, GLASGOW.

FIRST FLOOR PLAN

The total accommodation is for sixty nurses and domestics, and each nurse has a well-furnished room for herself. The electric light is used; open fire-places are provided throughout, and hot-water coils can be brought into action when the temperature requires their use.

The architects were Messrs. Thomson and Sandilands, of Glasgow. The cost is not stated.

## Figures and Tables

**Figure f1:**
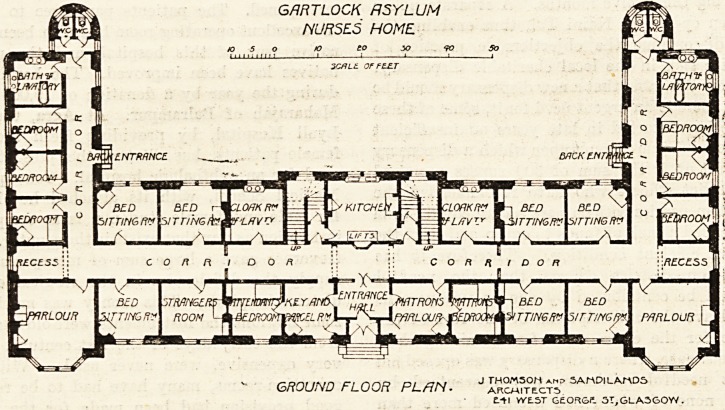


**Figure f2:**